# Age and Flourishing Mental Health: The Role of Rumination on Well-Being

**DOI:** 10.1093/geroni/igaf122.3272

**Published:** 2025-12-31

**Authors:** Seerat Kang, Meghan Elliot, Susan Charles, Joseph Mikels

**Affiliations:** University of California, Irvine, Irvine, California, United States; University of California, Irvine, Irvine, California, United States; University of California, Irvine, Irvine, California, United States; DePaul University, Chicago, Illinois, United States

## Abstract

A growing number of studies find that older age is often related to higher levels of emotional well-being despite marked declines in cognitive and physical function. Although the positivity effect is not an emotion regulation strategy, an age-related preference to attend to more positively valenced affective experiences may contribute to higher levels of well-being. The current study hypothesized that age is related to higher levels of emotional well-being, and that this association is due to age differences in the tendency to dwell on negative emotions (rumination) and not specific coping strategies. Adults (N = 393) ranging from 18 to 83 years old completed measures of emotional wellbeing (positive & negative affect; flourishing mental health), rumination, and coping strategies (avoidant; emotion-focused, problem-focused, and social support). Age was not significantly associated with higher rates of positive affect. However, older age was related to higher levels of flourishing mental health (B(SE) = .012(.003), p = .0002), even with the addition of coping strategies in the model. With the addition of rumination to the model, rumination accounted for 52% of the association between age and flourishing mental health (B(SE) = .005(.003), p = .05), suggesting higher levels of psychological well-being in older age are related to decreased ruminative tendencies rather than previously posited increases in positive affective experiences.

